# Highly sensitive and multiplexed analysis of CpG methylation at single-base resolution with ligation-based exponential amplification†
†Electronic supplementary information (ESI) available. See DOI: 10.1039/c4sc03135k



**DOI:** 10.1039/c4sc03135k

**Published:** 2014-12-11

**Authors:** Fengxia Su, Limei Wang, Yueying Sun, Chenghui Liu, Xinrui Duan, Zhengping Li

**Affiliations:** a Key Laboratory of Medicinal Chemistry and Molecular Diagnosis , Ministry of Education , College of Chemistry and Environmental Science , Hebei University , Baoding 071002 , Hebei Province , P. R. China . Email: lzpbd@snnu.edu.cn ; Fax: +86 29 81530859 ; Tel: +86 29 81530859; b Key Laboratory of Applied Surface and Colloid Chemistry , Ministry of Education , Key Laboratory of Analytical Chemistry for Life Science of Shaanxi Province , School of Chemistry and Chemical Engineering , Shaanxi Normal University , Xi'an 710062 , Shaanxi Province , P. R. China

## Abstract

DNA methylation is a primary epigenetic mechanism for transcriptional regulation during normal development and the occurrence of diseases, including cancers. DNA methylation has been increasingly utilized as a biomarker for cancer detection and differential diagnosis. Generally, one type of cancer is associated with several CpG methylation sites and detection of multiplexed CpG methylation can greatly improve the accuracy of cancer diagnosis. In this paper, we have developed a novel ligase chain reaction (LCR)-based method for multiplexed detection of CpG methylation in genomic DNA at single-base resolution. By rationally designing the two pairs of DNA probes for LCR, the bisulfite-treated methylated DNA target can be exponentially amplified by thermal cycling of the ligation reaction, in which one-base mismatch can be discriminated against, and thus high sensitivity and specificity for the detection of DNA methylation can be achieved. The LCR-based method can accurately determine as low as 10 aM methylated DNA fragment and 10 ng methylated genomic DNA. 0.1% methylated DNA can be detected in the presence of a large excess of unmethylated DNA. Moreover, by simply encoding one of the DNA probes in the LCR with a different length of poly(A) for detection of methylation at different CpG sites, the CpG methylation at different sites can produce LCR products with different lengths, and thus, can be simultaneously detected with one-tube LCR amplification and separation by capillary electrophoresis.

## Abstract 2

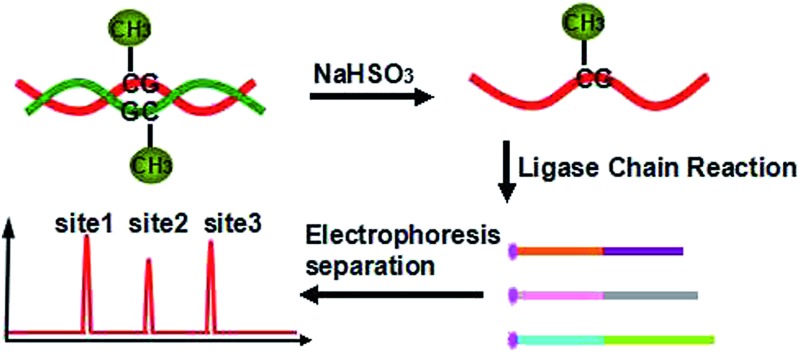
Multiple CpG methylation can be accurately detected in one-tube ligase chain reaction (LCR) amplification with high sensitivity and specificity.

## Introduction

In mammalian cells, abundant DNA methylation at the 5-position of cytosine within the CpG dinucleotides is the most well characterized epigenetic modification, which plays a crucial role in genomic imprinting, X chromosome inactivation, and regulation of gene expression during development and diseases, cancers in particular.^1,2^ Aberrant DNA methylation frequently occurs at gene promoters during cancer progression.^3^ Hypermethylation at the promoter of tumor-suppressor genes and hypomethylation at the promoter of oncogenes, which are respectively associated with inappropriate gene silencing of tumour-suppressor genes and overexpression of oncogenes, are considered to be important drivers of tumorigenesis.^3,4^ More complicated misregulation of a gene can also be caused by aberrant methylation, for example, the hypermethylation of a p53 binding site can lead to overexpression of oncogenes.^5^

The earliest used approach for genome-wide profiling of DNA methylation sites was the restriction landmark genomic scanning (RLGS).^6^ The microarray-based techniques have provided important insights into the association between the methylation patterns and cancer types.^7^ More recently, advanced “next-generation DNA sequencing” technologies have revolutionized our capabilities for the global mapping of methylation patterns at single-base resolution, providing new insights into the regulation roles of DNA methylation in human cancers.^8^ The comparative analyses of DNA methylation patterns between various malignant and normal tissue samples have revealed that one type of cancer is associated with hypermethylation of the CpG islands in promoter regions of multiple genes, which can be regarded as potential biomarkers for early cancer diagnosis and prognosis.^4^ It has also been demonstrated that the simultaneous detection of multiplexed CpG methylation can greatly improve the accuracy of cancer diagnosis.^9^ The advances in the identification of DNA methylation patterns have highlighted the need to develop multiplexed and sensitive methods for the accurate detection of DNA methylation at single-base resolution to be routinely used for cancer diagnosis and for fundamental research into the biological functions of DNA methylation.

Generally, there are three approaches employed to discriminate methylated cytosine and unmethylated cytosine in genomic DNA: affinity enrichment, endonuclease digestion, and bisulfite conversion.^10^ The first approach captures the methylated DNA fragments with methyl-binding domain proteins or antibodies specific to methylated cytosine, and is usually of low resolution for the determination of the methylated sites in DNA.^8^ In the second approach, methylation-sensitive restriction enzymes are used to distinguish between methylated and unmethylated recognition sites in genomic DNA, which can provide a straightforward readout of the methylated sites, but is restricted by the limited availability of enzyme-recognition sites.^11^ The last approach, bisulfite conversion, is based on the treatment of DNA with sodium bisulphite under denaturing conditions, in which the cytosines can be reproducibly converted into uracils but methylcytosines are left unchanged.^12^ The bisulfite treatment can effectively turn an epigenetic difference into a sequence difference in genomic DNA, and it is now regarded as the gold-standard method of detecting cytosine methylation.^8^ In addition to the discrimination of methylated cytosine from cytosine, the sensitivity of the detection of methylated cytosine in genomic DNA is also imperative. In order to achieve enough sensitivity to detect methylated cytosine in low-quantity biological samples, DNA amplification is generally required and polymerase chain reaction (PCR) is most widely used in the investigation of single-locus methylation changes. Combined with different approaches to discriminate methylated and unmethylated cytosine, a wealth of PCR-based methods for the sensitive detection of DNA methylation have been developed. However, most of these methods require labor intensive post PCR detection techniques and/or expensive equipment, such as gel electrophoresis,^13^ radioactive single nucleotide primer extension (SNuPE),^14^ mass spectrometry,^15,16^ high-resolution melting analysis (HRMA),^17^ fluorescence resonance energy transfer (FRET),^18,19^ luciferase-fused zinc finger protein-based bioluminescence,^20^ quartz crystal microbalance (QCM),^21^ and combined bisulfite restriction analysis (COBRA).^22^ The fluorescence-based real-time PCR technologies, including MethyLight,^11^ HeavyMethyl,^23^ and quantitative analysis of methylated alleles (QAMA),^24^ require no further manipulations after the PCR step and can achieve quantitative results with high sensitivity. Although the PCR-based methods have considerably enhanced our ability to detect cytosine methylation in genomic DNA, some challenges still remain: (1) the PCR-based system is susceptible to false positive results, which is a major obstacle when used in diagnostic laboratories,^25^ (2) DNA degradation occurs during bisulfite treatment owing to oxidative damage, which can bring about misleading PCR results,^26^ and (3) the PCR-based methods are difficult to use for the simultaneous detection of multiple CpG methylation sites at different loci with one-tube PCR amplification. More recently, some PCR-free methods for the direct detection of DNA methylation have also been investigated, such as immuno-recognition-based colorimetry^26^ and electrochemiluminescence,^27^ and eMethylsorb.^28^ However, these methods generally have relatively low sensitivities which are not enough for the detection of DNA methylation in a genomic DNA sample.

Ligase chain reaction (LCR) is an alternative to PCR,^29^ in which two pairs of DNA probes, which hybridize at adjacent positions to the complementary strand of a target DNA sequence, are ligated by a thermostable ligase. With thermal cycles, multiple rounds of denaturation, annealing and ligation result in the exponential amplification of the target DNA. Due to the high specificity of DNA ligase to discriminate between a mismatched and a complementary DNA helix, LCR can directly genotype single nucleotide polymorphisms (SNPs) in genomic DNA.^30–32^

In this paper, we have developed a novel LCR-based methylation assay. Through the amplification of bisulfite-treated DNA targets with the designed LCR, methylated cytosine can be simultaneously recognized at single-base resolution and detected with high sensitivity. The methylated DNA target can be determined as low as 10 aM and 0.1% methylated DNA can be detected in the presence of large amounts of unmethylated DNA. More importantly, by encoding the DNA probes with different lengths for different methylated CpG sites, the different methylated CpG sites can yield LCR products with different lengths, which can be separated using capillary electrophoresis (CE). Thus, the multiplexed DNA methylation can be simultaneously determined with a one-tube LCR reaction.

## Experimental methods

### Materials and apparatus

Taq DNA ligase and CpG Methyltransferase (M.SssI) were purchased from New England Biolabs. Salmon sperm DNA was purchased from Invitrogen (USA). All water used in this study was sterilized and deionized. HPLC purified DNA probes and ULTRAPAGE purified DNA targets were obtained from Shanghai Sangon Biotech (Shanghai, China). The sequences of all synthetic oligonucleotides used in the study were listed in Table S1 (ESI†). TIANamp Genomic DNA Kit (TIANGEN Biotechnologies) was used to extract genomic DNA from human whole blood. EpiTect® Bisulfite kit was acquired from Qiagen (Germany). Hi-Di™ Formamide and GeneScan™ 120 LIZ® Size Standard were purchased from Applied Biosystems (USA). All other reagents were of analytical reagent grade and were used as purchased without further purification. The LCR reaction was carried out in a Biometra Thermocycler (Germany). LCR products were separated and detected with an ABI PRISM® 310 Genetic Analyzer (USA). A TU1901 UV-Vis spectrophotometer (Beijing Purkinje General Instrument Co. Ltd) was employed for quantification of genomic DNA.

### Genomic DNA extraction and methylation

Human genomic DNA was extracted from the whole blood of a healthy volunteer in our laboratory using a TIANamp Genomic DNA Kit. The extracted genomic DNA was quantified from the absorption at 260 nm with a UV-Vis spectrophotometer. Then the genomic DNA was divided into two equal parts. The first part was methylated with the treatment of the M.SssI according to the following steps, while the other part was not treated. The extracted genomic DNA (2 μg) was mixed with 0.64 mM *S*-adenosylmethionine (SAM) and 20 U M.SssI in buffer (50 mM NaCl, 10 mM Tris–HCl, 10 mM MgCl_2_, 1 mM DTT, pH 7.9 @ 25 °C) with a final volume of 20 μL and incubated at 37 °C for 5 h. Then 0.8 μL 16 mM SAM and 20 U M.SssI were added into the mixture and incubated at 37 °C for 12 h to ensure complete methylation. Afterward, the mixture was heated to 65 °C for 20 min to inactivate the M.SssI. The methylated and unmethylated genomic DNA were treated with sodium bisulfite and purified using an EpiTech® Bisulfite kit according to the manufacturer's protocols. The bisulfite-treated genomic DNA was finally quantified by the absorption at 260 nm.

### LCR

Probe A_M_ and probe B_M_ (each final concentration of 10 nM) were mixed with 3 μg salmon sperm DNA and an appropriate amount of target DNA, and heated to 95 °C for 5 min. Then a mixture of 4 U Taq DNA ligase and the ligase buffer (20 mM Tris–HCl, 25 mM KAc, 10 mM Mg(Ac)_2_, 10 mM DTT, 1 mM NAD, 0.1% Triton X-100, pH 7.6 @ 25 °C) was added to the hot mixture at 80 °C and ten thermal cycles were performed (each cycle consisted of 95 °C for 30 s and 68 °C for 2 min). Afterward, probe A′_M_ and probe B′_M_ (each final concentration is 10 nM) were added into the mixture at 80 °C and the LCR reaction was carried out with a further 30 thermal cycles of 95 °C for 30 s and 68 °C for 2 min.

### CE separation and detection of the LCR products

The separation and detection of the LCR products was performed on an ABI PRISM® 310 Genetic Analyzer. The LCR products were diluted 10-fold with water and 1 μL of the diluent was mixed with 18.8 μL Hi-Di™ Formamide and 0.2 μL GeneScan™ 120 LIZ® Size Standard. The mixture was heated at 95 °C for 5 min and cooled on ice for 10 min. A 47 cm × 50 μm capillary and POP-4 polymer (purchased from Applied Biosystems) were used for the CE separation of the LCR products. The settings of the 310 Genetic Analyzer were based on the manufacturer's instructions. The parameters for each run are: an injection time of 20 s, a voltage of 10 kV, and a run time of 24 min at 60 °C. The experimental data were analyzed using the GeneMapper 4.1 software (Applied Biosystems) and the positions and areas of the peaks were determined.

## Results and discussion

### Principle of LCR-based multiplexed CpG methylation assay

**Fig. 1:**
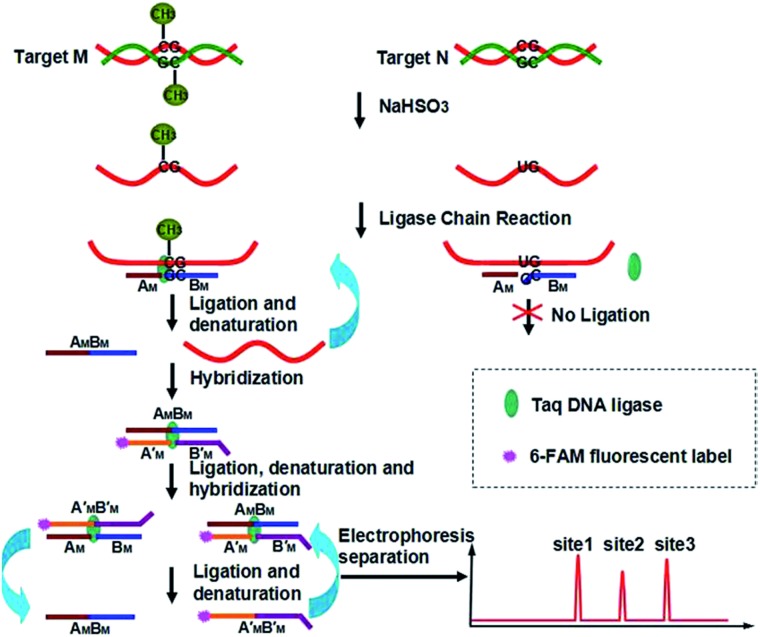
Schematic representation of the LCR-based multiplexed CpG methylation assay.

Our new strategy for the detection of multiplexed CpG methylation in genomic DNA is schematically illustrated in Fig. 1. Briefly, after denaturation, the methylated DNA target and unmethylated DNA target are simultaneously treated with sodium bisulfite, which reproducibly changes unmethylated cytosines to uracils (corresponding to thymine in DNA) but leaves methylated cytosines unchanged. In order to specifically detect a methylated CpG site in genomic DNA, probe A_M_ and probe B_M_ are employed to adjacently hybridize to the specific sequence of genomic DNA at the methylated CpG site, in which probe A_M_ is modified with a phosphate group at its 5′ end and the base at the 3′ end of probe B_M_ is guanine, complementary to methylated cytosine, so that the probe A_M_ and probe B_M_ can be ligated by the catalysis of Taq DNA ligase to form DNA strand A_M_B_M_. The guanine at the 3′ end of probe B_M_ is not complementary to uracil (or thymine) in the unmethylated CpG site, so no ligation occurs. After denaturation at 95 °C and hybridization at 68 °C, the methylated DNA can be sequentially used as the template to ligate probe A_M_ and probe B_M_. On the other hand, probe A′_M_ and probe B′_M_, in which probe A′_M_ is labeled with FAM at the 5′ end and probe B′_M_ is encoded with different lengths of poly(A) at the 3′ end for different methylated CpG sites and also modified with a phosphate group at the 5′ end, will hybridize to the DNA strand A_M_B_M_ and then are ligated to form double strand (ds) DNA A_M_B_M_/A′_M_B′_M_. Accordingly, with the thermal cycles of denaturation, hybridization and ligation, the DNA strands A_M_B_M_ and A′_M_B′_M_ will be used as the templates to hybridize and ligate probe A′_M_ and probe B′_M_, and probe A_M_ and probe B_M_, respectively. The ligation products from one round can become the templates for the next round of ligation, thus forming the ligation chain reaction (LCR), which can produce the ligation products in an exponential fashion by repeated thermal cycling. Moreover, for different methylated CpG sites, the LCR will produce FAM-labeled DNA strands A′_M_B′_M_ with different lengths. After denaturing with formamide, the LCR products are separated using CE and detected with laser induced fluorescence (LIF). Therefore, different methylated CpG sites can be simultaneously detected in a one-tube LCR amplification.

### Evaluation of the specificity of the LCR-based assay

Hypermethylation of CpG sites in the promoter region of the metalloproteinase-3 (TIMP-3) gene has a close relationship with colon cancer.^33^ Three synthetic DNA fragments in the promoter region of TIMP-3 containing the different CpG sites are used as the model targets, whose sequences correspond to the bisulfite-treated methylated DNA. The synthetic DNA targets (see the sequences in Table S1, ESI†) containing CpG site 1, site 2, and site 3 are respectively defined as target M_1_, target M_2_, and target M_3_. The probes A_M_1__, B_M_1__ and 
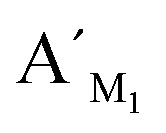
, 
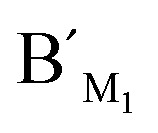
, probes A_M_2__, B_M_2__ and 
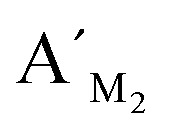
, 
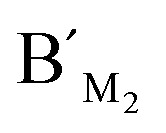
, and probes A_M_3__, B_M_3__ and 
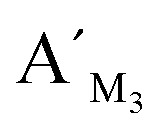
, 
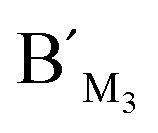
, are specific to the target M_1_, target M_2_, and target M_3_, respectively. The synthetic DNA target N_1_ corresponds to the bisulfite-treated unmethylated DNA containing CpG site 1, in which the uracil is substituted by thymine.^34^ The probes A_N_1__, B_N_1__ and 
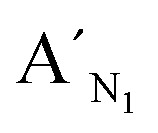
, 
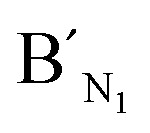
 are designed to be specific to the target N_1_.

**Fig. 2:**
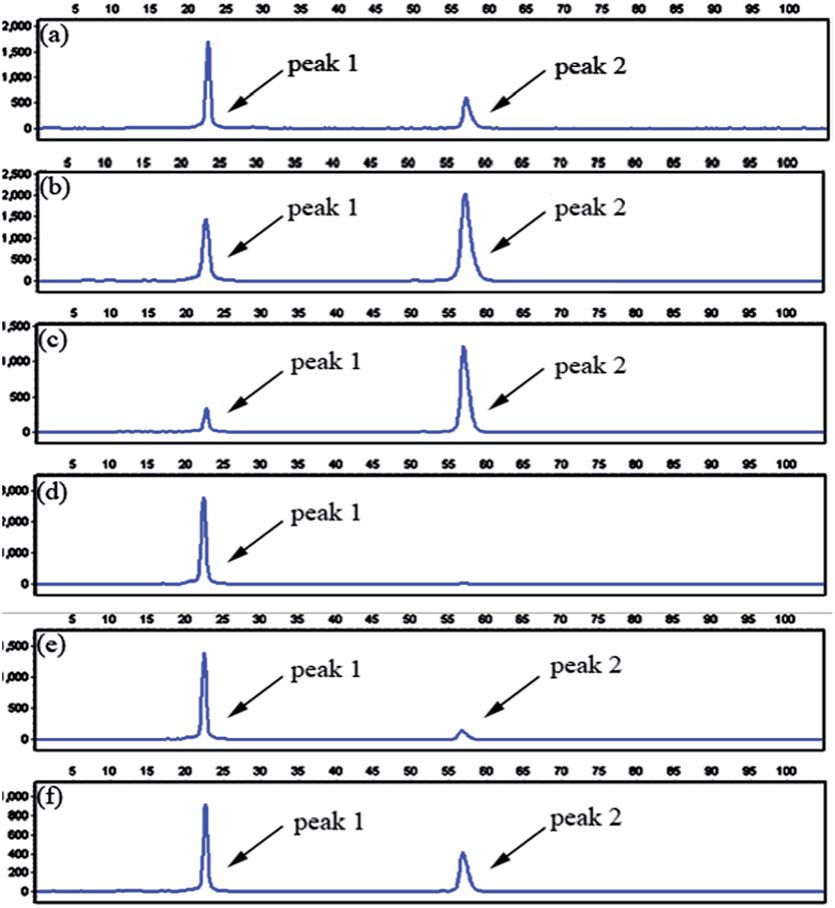
Electropherograms for the detection of target M_1_ and target N_1_ with the LCR-based assay by using target M_1_-specific probes (probe A_M_1__, B_M_1__, and 
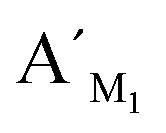
, 
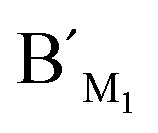
). (a) 100 aM target M_1_, (b) 1 fM target M_1_, (c) 10 fM target M_1_, (d) 10 fM target N_1_, (e) 100 fM target N_1_, (f) 1 pM target N_1_. The abscissa axis in the electropherograms represents the retention time and the longitudinal axis represents the relative fluorescence signal. Peak 1 is the signal of probe 
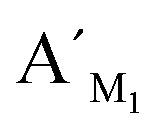
 and peak 2 is attributed to the LCR products. The LCR-based assay is performed according to the procedures described in the experimental methods.

**Fig. 3:**
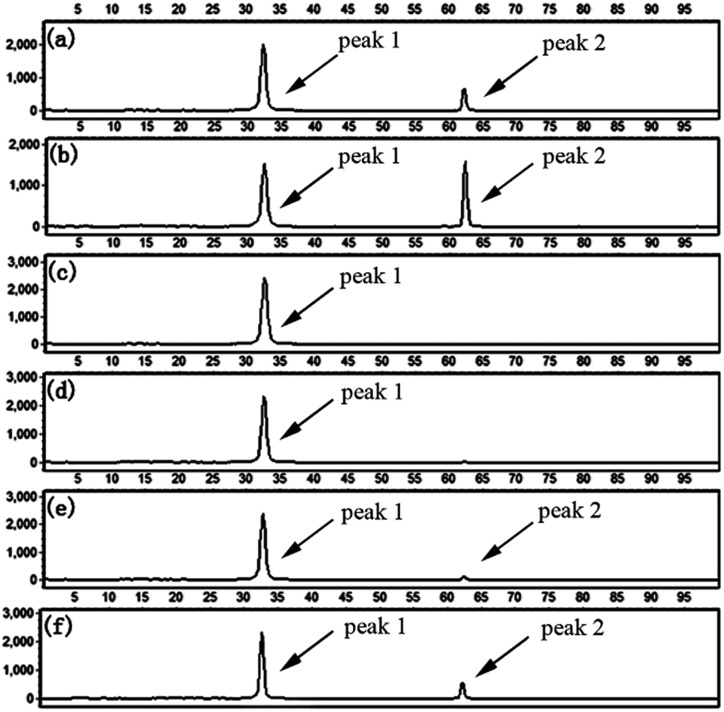
Electropherograms for the detection of target N_1_ and target M_1_ with the LCR-based assay by using target N_1_-specific probes (probe A_N_1__, B_N_1__, and 
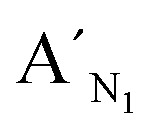
, 
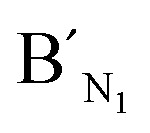
). (a) 100 aM target N_1_, (b) 1 fM target N_1_, (c) 100 aM target M_1_, (d) 1 fM target M_1_, (e) 10 fM target M_1_, (f) 100 fM target M_1_. The experimental conditions are the same as described in the caption of Fig. 2.

To evaluate the specificity of the proposed LCR-based assay, we firstly perform the LCR amplification by using target M_1_-specific probes to detect target M_1_ and target N_1_. As shown in Fig. 2, 100 aM, 1 fM and 10 fM target M_1_ can produce well-defined signals of LCR products, while 10 fM target N_1_ only produce a negligible signal. A large excess of target N_1_ can give rise to a detectable signal, which can be increased with increasing the concentration of target N_1_. However, the signal produced by 100 fM target N_1_ is less than that produced by 100 aM target M_1_, and the signal produced by 1 pM target N_1_ is less than that for 1 fM target M_1_. On the other hand, we also evaluate the specificity of the LCR-based assay by using target N_1_-specific probes to detect target N_1_ and target M_1_. As depicted in Fig. 3, the well-defined signals of the LCR products can be observed for detection of 100 aM and 1 fM target N_1_. At the same time, no detectable signal can be observed for 100 aM and 1 fM target M_1_. 10 fM and 100 fM target M_1_ can give rise to a very small signal of LCR products. The signal produced by 100 fM target M_1_ is less than that produced by 100 aM target N_1_. Therefore, the selectivity for detection of target M_1_ and target N_1_ with the LCR-based assay is greater than 1000-fold.

**Fig. 4:**
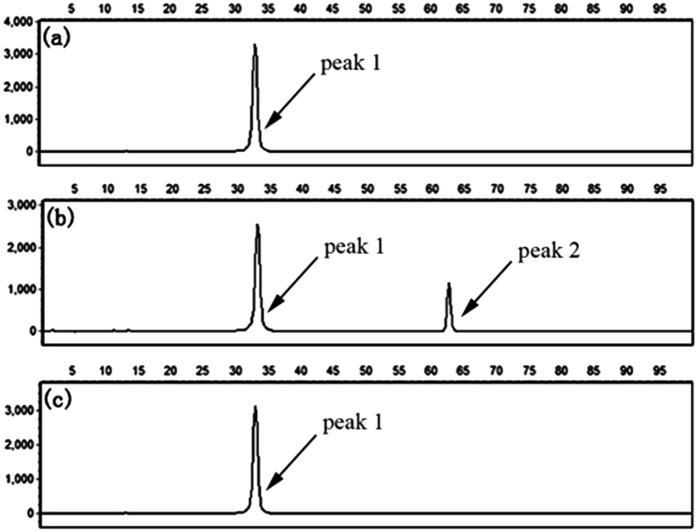
Electropherograms for the detection of methylated and unmethylated genomic DNA with the LCR-based assay by using target N_1_-specific probes (probe A_N_1__, B_N_1__, and 
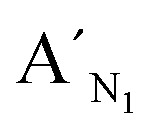
, 
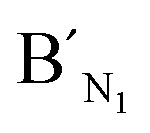
). (a) Blank, (b) 10 ng unmethylated genomic DNA, (c) 10 ng methylated genomic DNA. The experimental conditions are the same as described in the experimental methods. Blank was detected with the same procedures but without genomic DNA target.

**Fig. 5:**
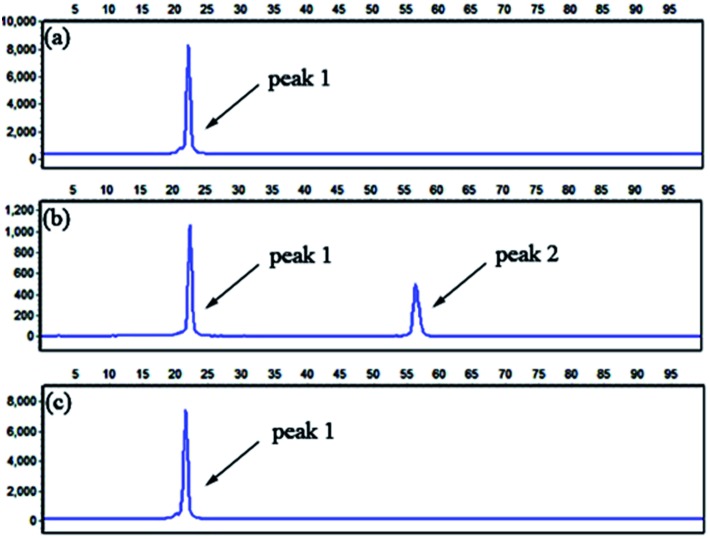
Electropherograms for the detection of methylated and unmethylated genomic DNA with the LCR-based assay by using target M_1_-specific probes (probe A_M_1__, B_M_1__, and 
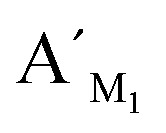
, 
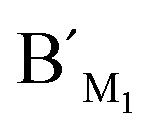
). (a) Blank, (b) 10 ng methylated genomic DNA, (c) 10 ng unmethylated genomic DNA. The experimental conditions are the same as described in the caption of Fig. 4.

To further confirm the specificity of the LCR-based assay, we apply the assay to detect methylated and unmethylated genomic DNA. The genomic DNA extracted from the whole blood of a healthy volunteer with informed consent serves as the unmethylated genomic DNA, which is treated with bisulfite under denaturing conditions, and then is detected with the LCR-based assay by using target N_1_-specific probes. All the experiments using genomic DNA were approved by the ethical committee of Hebei University and performed in compliance with the relevant laws and institutional guidelines. As demonstrated in Fig. 4a and b, in the absence of the genomic DNA, no signal of LCR products can be detected, indicating the very low background interference present in the LCR-based assay. The well-defined signal of LCR products can be observed to detect 10 ng unmethylated genomic DNA. Meanwhile, the genomic DNA is treated with CpG methyltansferase (M.SssI) and then served as the methylated genomic DNA. After bisulfite-treatment, as shown in Fig. 4c, the same amount of methylated genomic DNA doesn't produce a detectable signal by using target N_1_-specific probes to perform the LCR, which demonstrates the good selectivity for detection of the unmethylated genomic DNA. The results can also be used to determine that the extracted genomic DNA is unmethylated in the detected sequence region. On the other hand, the bisulfite-treated methylated genomic DNA is also detected with the LCR-based assay by using target M_1_-specific probes. One can see from Fig. 5b that bisulfite-treated methylated genomic DNA (10 ng) can be well detected. While, as shown in Fig. 5c, no detectable signal can be observed to detect the unmethylated genomic DNA, indicating the high specificity of the LCR-based assay for detection of methylated genomic DNA. These results have also demonstrated that the LCR-based assay can directly recognize the CpG methylation in genomic DNA samples at one-base resolution.

### Analytical performance of the LCR-based assay

**Fig. 6:**
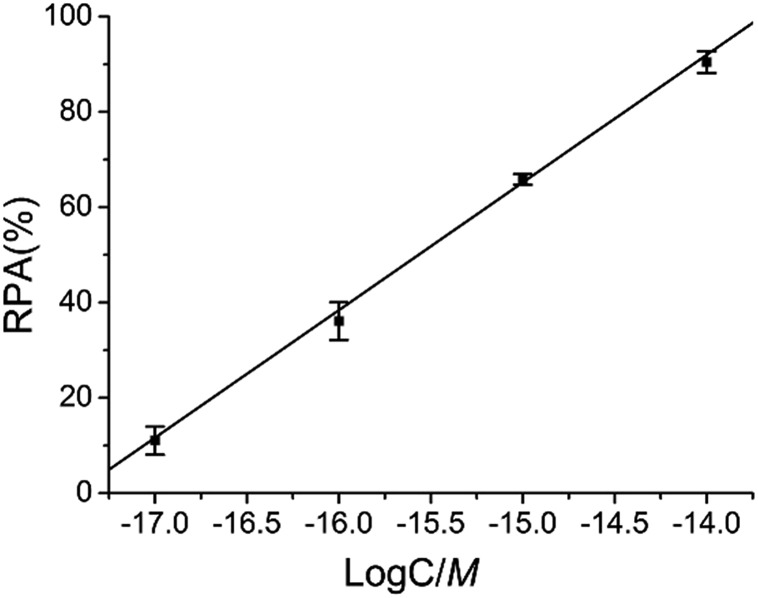
The linear relationship between RPA (%) and the log of target M_1_ concentration (M) in the LCR-based assay. The concentration of target M_1_ is 10 aM, 100 aM, 1 fM, and 10 fM, respectively. Error bars are estimated from the standard deviation of three repeat measurements. The LCR-based reaction is carried out according to the standard procedures in the experimental methods.

To assess the analytical performance of the LCR-based assay, a series of dilution of synthetic target M_1_ are determined with the LCR-based assay by using target M_1_-specific probes. As shown in Fig. S1 (ESI†), the peak height and peak area of the LCR products gradually increase with the increasing concentration of target M_1_, while those of the FAM-labeled probe 
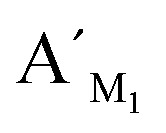
 correspondingly decrease. As low as 10 aM target M_1_ can be accurately determined with the LCR-based assay. In order to diminish the analysis bias resulting from the variation of different runs of CE-LIF detection, the relative peak area (RPA) is adopted for quantitation of the methylated DNA, which can be calculated as follows:
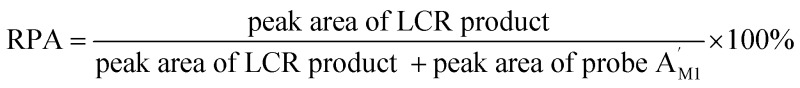


As demonstrated in Fig. 6, there is an excellent linear relationship between RPA (%) and the logarithm (log) of target M_1_ concentration in the range of 10 aM to 10 fM. The correlation equation is RPA (%) = 466.76 + 26.77 log *C* (M) and the corresponding correlation coefficient *R* is 0.999, indicating that the LCR-based assay can be applied for the quantitative determination of methylated DNA and has a wide linear detection range over 3 orders of magnitude.

We also perform the LCR-based assay to detect target N_1_ at different concentrations by using target N_1_-specific probes. As depicted in Fig. S2 and S3 (ESI†), target N_1_ can also be determined in the concentration range from 10 aM to 10 fM. The correlation equation is RPA (%) = 366.84 + 21.47 log *C* (M) with a correlation coefficient of *R* = 0.999.

The ideal strategy for tumor diagnosis at its early stage is to accurately detect the molecular biomarkers such as DNA methylation in body liquids such as blood, where large amounts of normal DNA exists. Therefore, it is significantly important to be able to detect small amounts of methylated DNA in the presence of a large excess of unmethylated DNA. Taking advantage of the high specificity and sensitivity, we test the capability of the LCR-based assay to detect methylated DNA in the presence of unmethylated DNA. The target M_1_ and target N_1_ are mixed with a total concentration of 10 fM as the samples, in which the target M_1_ proportions are 0%, 0.1%, 1%, 10%, and 100%, respectively. The mixture samples are detected with the LCR-based assay by using target M_1_-specific probes. The electropherograms for detection of these mixture samples are shown in Fig. S4 (ESI†). One can see from Fig. S4 and 7 that as low as 0.1% methylated DNA can be detected with the LCR-based assay.

**Fig. 7:**
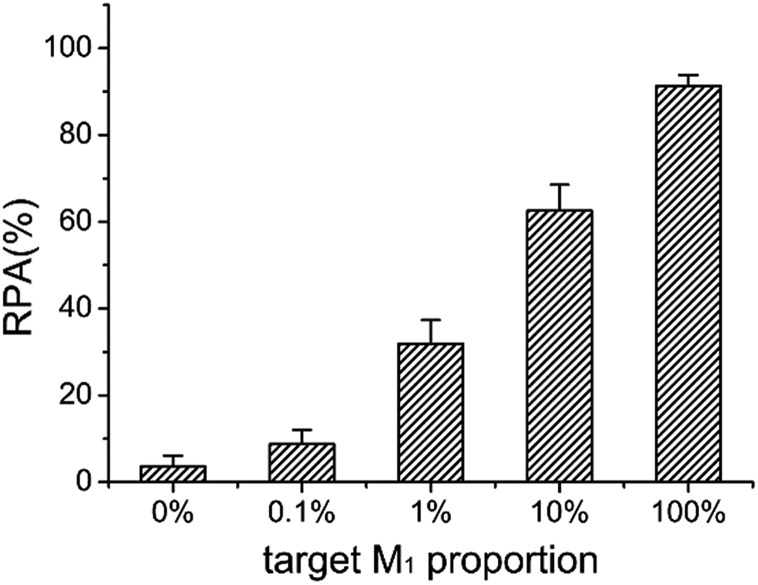
The RPA (%) produced by target M_1_ with different proportions in the mixture of target N_1_ and target M_1_ by using target M_1_-specific probes (probe A_M_1__, B_M_1__, and 
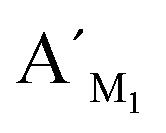
, 
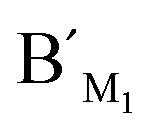
) in the LCR-based assay. The target M_1_ and target N_1_ are mixed with a fixed total concentration of 10 fM, and the target M_1_ proportions are 0%, 0.1%, 1%, 10%, and 100%, respectively. The LCR-based reaction is carried out according to the procedures in the experimental methods.

### Multiplexed CpG methylation detection

**Fig. 8:**
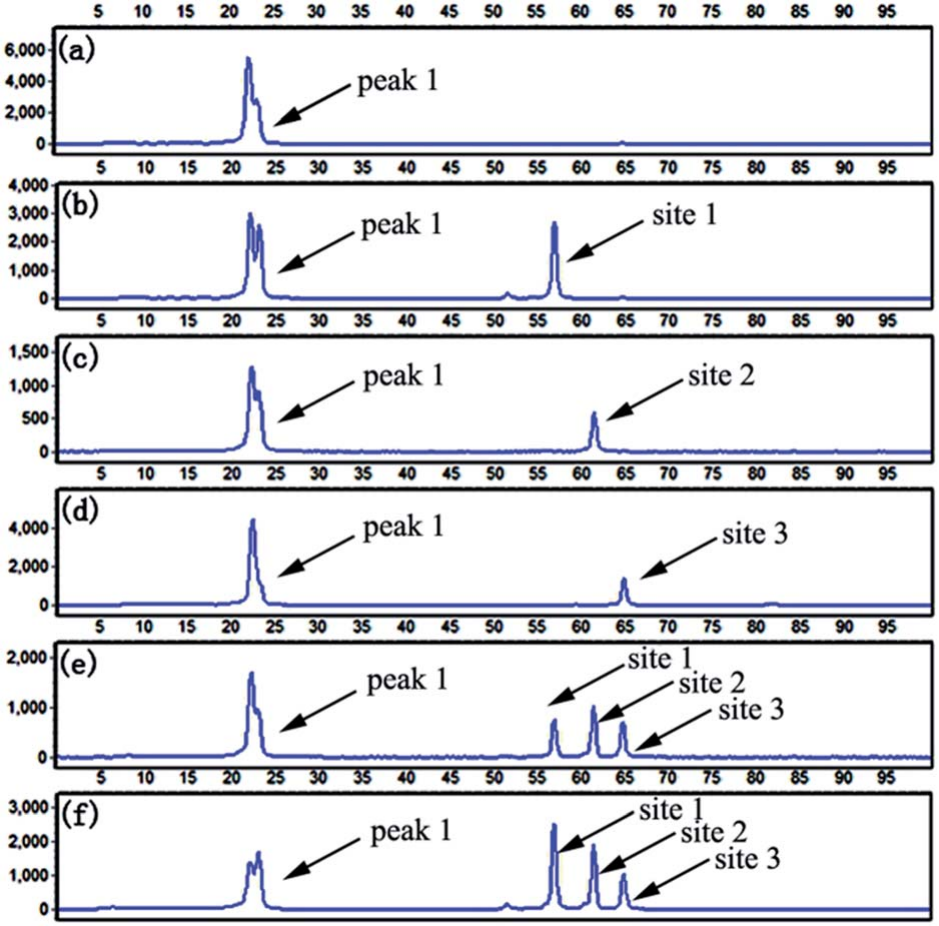
Electropherograms for detection of multiplexed CpG methylation sites with the LCR-based assay by using the mixture of probes, which are corresponding to target M_1_, target M_2_ and target M_3_. The detection samples are (a) blank, (b) 10 fM target M_1_ only, (c) 10 fM target M_2_ only, (d) 10 fM target M_3_ only, (e) 10 fM target M_1_, target M_2_, target M_3_, each, and (f) 40 ng methylated genomic DNA. Peak 1 is the signal of probe 
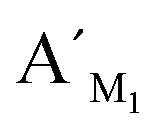
, probe 
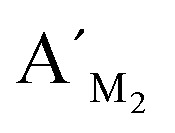
, and probe 
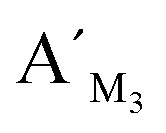
, and the peaks of site 1, site 2, site 3 are produced by the three detecting sites corresponding to target M_1_, target M_2_, and target M_3_, respectively. The experimental conditions are the same as described in the experimental methods.

To evaluate the multiplexing capability of the LCR-based assay, the DNA probes specific to target M_1_, target M_2_ and target M_3_ are mixed. Firstly, target M_1_, target M_2_, and target M_3_ are individually detected with the LCR-based assay by using the mixed probes. As depicted in Fig. 8b–d, the signals at different retention times for different targets can be observed. As demonstrated formerly, the probe B′_M_ is designed with different lengths for different methylated DNA targets. Accordingly, in the LCR products respectively produced from target M_1_, target M_2_, target M_3_, the length of FAM-labeled A′_M_B′_M_ is 61, 66 and 71 nucleotides (nts), respectively. Afterward, 10 fM target M_1_, target M_2_ and target M_3_ are mixed and detected with the LCR-based assay in one-tube LCR amplification by using the mixed probes. As shown in Fig. 8e, the FAM-labeled A′_M_B′_M_ in the LCR products respectively produced by different methylated DNA targets can be well separated and sensitively detected. By comparing the retention time of different peaks shown in Fig. 8b–e, it can be deduced that the size of FAM-labeled A′_M_B′_M_ in the LCR product of mixed methylated samples is identical to that produced by the individual methylated DNA target. To further test the practicality of the LCR-based assay, 40 ng methylated genomic DNA is analyzed with the LCR-based assay by using the mixed probes. One can see from Fig. 8f, the three well-defined signals corresponding to methylated CpG at site 1, site 2 and site 3 can be detected, indicating that the LCR-based assay can be well applied to multiplexed analysis of CpG methylation in genomic DNA samples.

## Conclusions

In conclusion, we have developed a LCR-based quantitative analysis of CpG methylation. The significant advantages of high sensitivity and specificity over conventional methods of methylation detection can be gained by the use of LCR amplification. Taking advantage of the high specificity of the LCR amplification, as low as 0.1% methylated DNA can be determined at single-base resolution in the presence of a large excess of unmethylated DNA. The DNA probes can be simply designed to adjacently hybridize to the DNA target at the position of the methylated cytosine. The LCR can accurately recognize the methylated sites, therefore, the methylated states will be directly read out by detecting the LCR products. The high sensitivity with a low background can be achieved with the LCR-based assay, in which as low as 10 aM methylated DNA target can be determined, corresponding to about 60 copies of methylated DNA molecule in a 10 μL solution. Finally, the LCR-based assay can easily realize the multiplexed analysis of CpG methylation by simply designing one DNA probe with different lengths for different methylated DNA targets. The length of the DNA probe can be modulated with poly(A), which does not effect the melting temperature between the DNA target and DNA probe and thus, does not affect the LCR amplification. In our proof of principle work, all the three methylated sites can be well determined with one-tube LCR amplification in both a synthetic sample and genomic DNA. Generally, one type of cancer is associated with several methylated CpG sites. Simultaneous detection of multiplexed methylated sites with a one-tube amplification reaction can greatly improve the accuracy of cancer diagnosis, as described formerly.^9^ Moreover, the amount of DNA available for cancer diagnosis is often limited. The LCR-based assay is also greatly significant for detection of multiplexed CpG methylation with seriously limited-quantity clinical samples. Therefore, we believe that the proposed LCR-based assay has great potential for the diagnosis of cancer and other methylation-related diseases by using CpG methylation as the molecular biomarker.
