# Supplementary material for: Highly sensitive and multiplexed analysis of CpG methylation at single-base resolution with ligation-based exponential amplification
Source: Chem Sci. 2014 Dec 11;6(3):1866–72. doi: 10.1039/c4sc03135k (PMC5494546; doi:10.1039/c4sc03135k)
Supplement: Supplementary file 1 [file SC-006-C4SC03135K-s001.pdf]

Electronic Supplementary Material (ESI) for Chemical Science.
This journal is © The Royal Society of Chemistry 2014

Electronic Supplementary Information

Highly Sensitive and Multiplexed Analysis of CpG Methylation at Single-base Resolution with Ligation-Based Exponential Amplification

Fengxia Su,[a] Limei Wang,[a] Yueying Sun,[a] Chenghui Liu,[b] Xinrui Duan,[b] and Zhengping Li *,[ab]

[a] Key Laboratory of Medicinal Chemistry and Molecular Diagnosis, Ministry of Education; College of Chemistry and Environmental Science, Hebei University, Baoding 071002, Hebei Province, P. R. China.

[b] Key Laboratory of Applied Surface and Colloid Chemistry, Ministry of Education; Key Laboratory of Analytical Chemistry for Life Science of Shaanxi Province, School of Chemistry and Chemical Engineering, Shaanxi Normal University, Xi'an 710062, Shaanxi Province, P. R. China

Email: lzpbd@snnu.edu.cn

List of Contents:

1. Table S1. The sequences of targets and probes used in the experiments.

2. The electropherograms for detection of target M_1 at different concentrations.

3. The analytical performance of the LCR-based assay for detection target N_1.

4. The electropherograms for detection of target M_1 in the mixture samples of target M_1 and target N_1.

1. Table S1. The sequences of targets and probes used in the experiments (5′-3′).

Targets and probes	Sequences
Target M_1	GGAGGTTAAGGTTGTTTCGTACGGTTCGGCGGGCGAGCGAGTTCGGGTTGTAGTAGTTT
Probe A_{M1}	Phosphate-CCGAACCGTACGAAACAACCTTAACCTCC
Probe B_{M1}	AAAAACTACTACAACCCGAACTCGCTCGCCCG
Probe A'_{M1}	FAM-AAGGAGGTTAAGGTTGTTTCGTACGGTTCGGC
Probe B'_{M1}	Phosphate-GGGCGAGCGAGTTCGGGTTGTAGTAGTTT
Target N_1	GGAGGTTAAGGTTGTTTTGTATGGTTTGGTGGGTGAGTGAGTTTGGGTTGTAGTAGTTT
Probe A_{N1}	Phosphate-CCAAACCATACAAAACAACCTTAACCTCC
Probe B_{N1}	AAAAACTACTACAACCCAAACTCACTCACCCA
Probe A'_{N1}	TAMRA-AAGGAGGTTAAGGTTGTTTTGTATGGTTTGGT
Probe B'_{N1}	Phosphate-GGGTGAGTGAGTTTGGGTTGTAGTAGTTT
Target M_2	TTTTTTTTTGGAGGGTCGATGAGGTAATGCGGTTTTGTTATTGGTTTGAGGGGGCGGGT
Probe A_{M2}	Phosphate-CATTACCTCATCGACCCTCCAAAAAAAAA
Probe B_{M2}	AAACCCGCCCCCTCAAACCAATAACAAAACCG
Probe A'_{M2}	FAM-AATTTTTTTTTGGAGGGTCGATGAGGTAATGC
Probe B'_{M2}	Phosphate-GGTTTTGTTATTGGTTTGAGGGGGCGGGTAAAAA
Target M_3	AGTTCGAGGCGGGGTTTTCGGGGGTTTAGCGTTATATTATTCGGTCGTTTAGGTAGCGG
Probe A_{M3}	Phosphate-CTAAACCCCCGAAAACCCCGCCTCGAACT
Probe B_{M3}	AACCGCTACCTAAACGACCGAATAATATAACG
Probe A'_{M3}	FAM-AAAGTTCGAGGCGGGGTTTTCGGGGGTTTAGC
Probe B'_{M3}	Phosphate-GTTATATTATTCGGTCGTTTAGGTAGCGGAAAAAAAAAA

Note: Target M_1, target M_2, and target M_3 are synthetized DNA fragments in the promoter region of TIMP-3 gene, which respectively contains the methylated CpG site 1, site 2, and site 3 (underlined in the sequence). The sequences of target M_1, target M_2, and target M_3 are corresponding to the methylated DNA sequences after bisulfite-treatment. The sequence of target N_1 is corresponding to

the unmethylated target M_1 sequence after bisulfite-treatment, in which uracil is substituted by thymine. The relationship between other targets and their probes is the same as that between target M_1 and their probes. The probe A_{M1}, B_{M1}, A'_{M1}, B'_{M1}, probe A_{N1}, B_{N1}, A'_{N1}, B'_{N1}, probe A_{M2}, B_{M2}, A'_{M2}, B'_{M2}, and probe A_{M3}, B_{M3}, A'_{M3}, B'_{M3} are specific to the target M_1, target N_1, target M_2, and target M_3, respectively.

2. The electropherograms for detection of target M_1 at different concentrations.

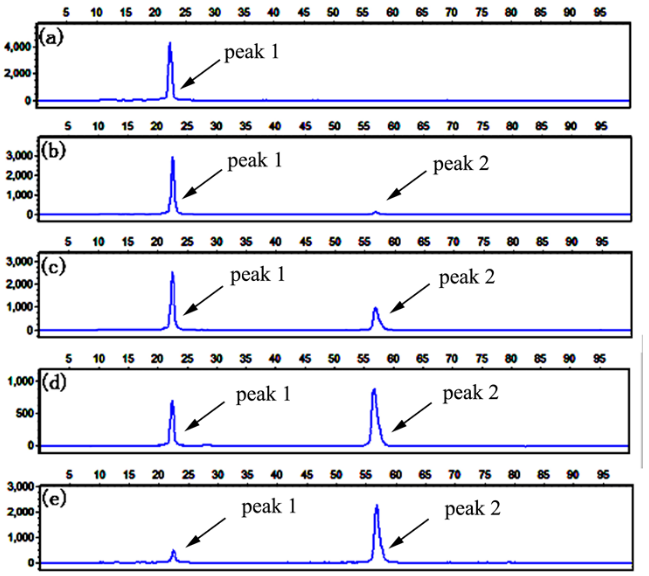

Fig. S1. The electropherograms for detection of target M_1 at different concentrations with the LCR-based by using target M_1-specific probes (probe A_{M1}, B_{M1}, A'_{M1}, and B'_{M1}). The concentration of target M_1 is (a) blank, (b) 10 aM, (c) 100 aM, (d) 1 fM, and (e) 10 fM, respectively. The abscissa axis in the electropherograms represents the retention time and the longitudinal axis represents the relative fluorescence signal. Peak 1 is the signal of probe A'_{M1} and peak 2 is produced by the LCR products. The LCR-based assay is performed according to the procedures described in the Experimental methods.

3. The analytical performance of the LCR-based assay for detection target N_1.

Besides the analytical performance evaluation for the detection of target M_1, we further

investigated the analytical performance of target N_1 in order to validate the capability of the LCR-based assay. A series of dilution of target N_1 were detected with the LCR-based assay by using target N_1-specific probes (probe A_{N1}, B_{N1}, A'_{N1}, and B'_{N1}). As shown in Fig. S2, the peak area of LCR products gradually increased with increasing the target N_1 concentration, while the peak area of FAM-labeled probe A'_{N1} decreased. After calculating the relative peak area (RPA) for each concentration of target N_1, as depicted in Fig. S3, a good linear relationship between RPA(%) and logarithm (log) of target N_1 concentration was obtained. The linear curve is fitted the equation of RPA (%) = 366.84 + 21.47 log C (M) and the corresponding correlation coefficient R is 0.999. The target N_1 can be accurately detected as low as 10 aM, with a linear range of over 3 orders of magnitude.

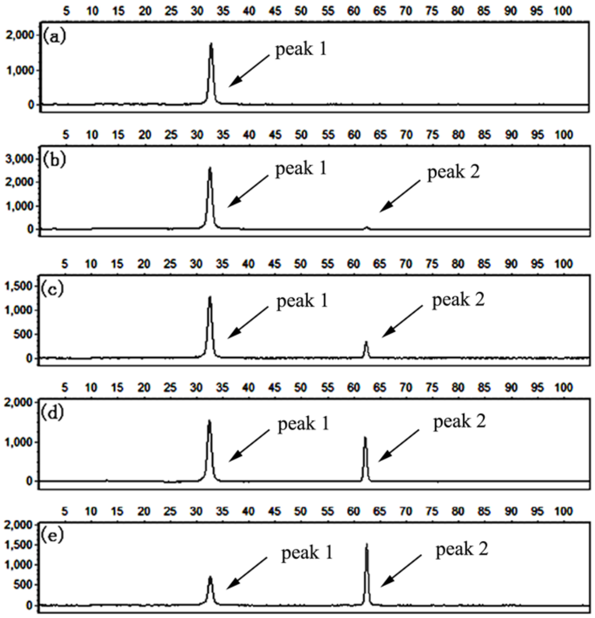

Fig. S2. The electropherograms for detection of target N_1 at different concentrations with the LCR-based by using target N_1-specific probes (probe A_{N1}, B_{N1}, A'_{N1}, and B'_{N1}). The concentration of target N_1 is (a) blank, (b) 10 aM, (c) 100 aM, (d) 1 fM, and (e) 10 fM, respectively. Peak 1 is the signal of probe A'_{N1} and peak 2 is produced by the LCR products. The experimental conditions are the same as described in Fig. S1.

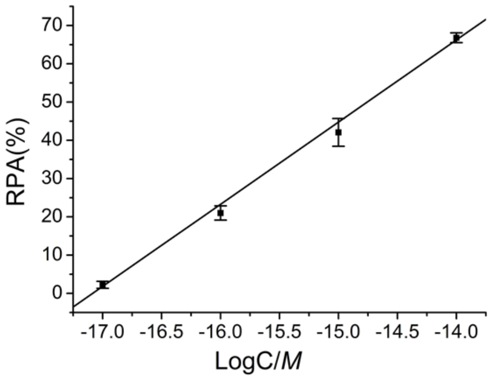

Fig. S3. The linear relationship between RPA(%) and log of target N_1 concentration (M) in the LCR-based assay. The concentration of target N_1 is 10 aM, 100 aM, 1 fM, and 10 fM, respectively. Error bars are estimated from the standard deviation of three repetitive measurements.

4. The electropherograms for detection of target M_1 in the mixture samples of target M_1 and target N_1.

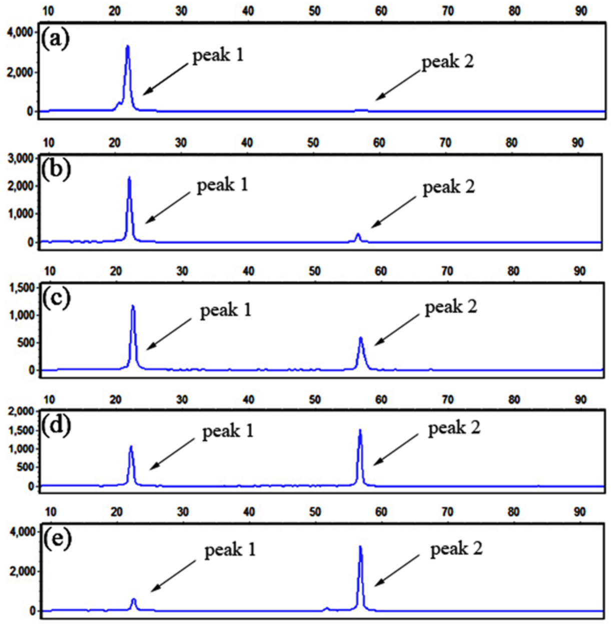

Fig. S4. The electropherograms for detection of target M_1 with different proportions in the mixture of target N_1 and target $M_1$with LCR-based assay by using target M_1-specific probes (probe A_{M1}, B_{M1}, A'_{M1}, and B'_{M1}). The target M_1 and target N_1 are mixed with a fixed total concentration of 10

fM, in which the proportion of target M_1 is 0%, 0.1%, 1%, 10%, and 100%, respectively. The peak 1 is the signal of probe A'_{M1} and the peak 2 is generated by the LCR products. The experimental conditions are the same as that described in Experimental methods.
